# Molecular and Translational Anti-Inflammatory Strategies After Abdominal and Liver Surgery: From Immunonutrition to Cell-Based Therapies

**DOI:** 10.3390/ijms27219727

**Published:** 2026-10-29

**Authors:** Barbara Pietrzyk, Wiktor Pierzchała, Jedrzej Mikolajczyk, Piotr Lis, Anna Heliasz, Bruno Kaczmarczyk

**Affiliations:** Department of Medical Biophysics, Faculty of Medical Sciences in Katowice, Medical University of Silesia, 40-055 Katowice, Poland

**Keywords:** abdominal surgery, postoperative inflammation, immunonutrition, microbiota modulation, nuclear factor erythroid 2-related factor 2

## Abstract

Postoperative inflammation influences recovery after abdominal and liver surgery. This narrative review critically synthesizes clinical and translational evidence on inflammation-modulating strategies using a structured search of PubMed, Scopus, Web of Science, and Cochrane Library for publications from 2015 to 2025. Case reports, pediatric studies, and publications lacking clinical or translational relevance were excluded. Immunonutrition and early enteral nutrition are the most established approaches, with benefits for postoperative recovery and infectious complications. Perioperative corticosteroids in liver resection and short-course antibiotics following adequate source control offer procedure- and indication-specific benefits. Emerging interventions, including microbiota modulation, N-acetylcysteine, activation of nuclear factor erythroid 2-related factor 2/heme oxygenase-1 signaling, and cell-based therapies, show biological promise but lack sufficient high-quality clinical evidence for routine implementation. Established strategies should be integrated into Enhanced Recovery After Surgery pathways according to patient risk and surgical context. Personalized management requires prospective validation of emerging therapies and candidate biomarkers, including interleukin-6, C-reactive protein, and heme oxygenase-1, with standardized assays and sampling schedules.

## 1. Introduction

The postoperative inflammatory response is a physiological reaction of the body to surgical trauma; however, its excessive activation may lead to systemic inflammatory response syndrome (SIRS) and sepsis, ultimately resulting in multiorgan failure. Contemporary therapeutic approaches focus not on simply suppressing inflammation, but on its precise modulation, targeting key molecular and immunological pathways [1]. A central component of this response is the excessive activation of the cytokine axis involving interleukin-1β, interleukin-6 (IL-6), and tumor necrosis factor-α (TNF-α), accompanied by oxidative stress and intestinal barrier dysfunction. These disturbances increase the risk of severe complications, prolong hospital stay, and worsen clinical outcomes [2,3,4,5,6,7]. Modern therapeutic interventions include immunomodulation through effects on cytokine pathways, redox balance (the nuclear factor erythroid 2-related factor 2 [NRF2]/heme oxygenase-1 [HO-1] system), the gut microbiota, and cellular immunity, particularly the function of regulatory T cells (Tregs) [8,9,10].

The liver occupies a unique position at the interface of metabolism, nutrition, and immune regulation. Continuous exposure to gut-derived nutrients, microbial products, and antigens through the portal circulation requires a tightly regulated balance between immune surveillance, inflammatory activation, and tolerance [11,12]. Hepatocytes and non-parenchymal cell populations, including Kupffer cells, liver sinusoidal endothelial cells, hepatic stellate cells, and resident lymphocyte subsets, collectively coordinate acute-phase responses, innate and adaptive immune signaling, metabolic homeostasis, and tissue repair [13]. Surgical injury, ischemia–reperfusion, and perioperative nutritional stress can disrupt this immunometabolic equilibrium, providing a biological rationale for therapeutic strategies that simultaneously target inflammatory signaling, redox homeostasis, intestinal barrier integrity, and nutritional support [14].

**Figure 1:**
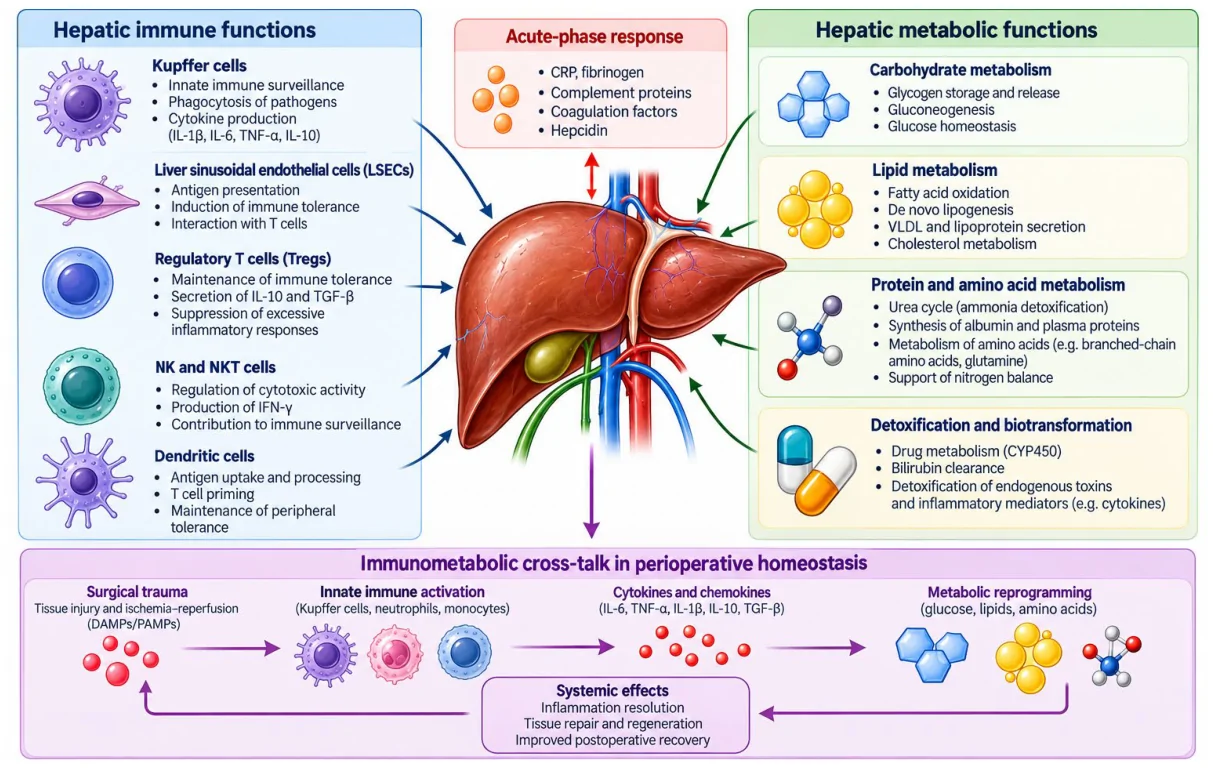
The liver: immunological and metabolic functions relevant to perioperative homeostasis [14].

The immunological and metabolic functions of the liver and their interplay in maintaining perioperative homeostasis are illustrated in Figure 1.

Increasing importance is also being attributed to cellular approaches, including therapies employing mesenchymal stem cells (MSCs) [15,16,17,18,19,20]. In addition, redox-targeted strategies involving NRF2 activation are being investigated as potential modulators of perioperative oxidative and inflammatory injury. Under conditions of oxidative stress, NRF2 escapes KEAP1-mediated degradation, translocates to the nucleus, and induces antioxidant response element-dependent transcription of cytoprotective genes, including HO-1, thereby enhancing endogenous antioxidant defenses and limiting redox-sensitive inflammatory signaling [21].

In clinical practice, immunonutrition [3,4,5], early enteral nutrition (EEN) [6,8,9], short-course antibiotic therapy following effective source control [22,23], and microbiota modulation [24] represent clinically relevant perioperative strategies in abdominal surgery.

Despite the increasing number of studies addressing individual immunomodulatory strategies, there remains a lack of integrated evidence clarifying their combined role in modulating the postoperative inflammatory response following abdominal surgery. Moreover, it is still unclear which interventions and associated biomarkers are most appropriate for precise patient stratification and the personalization of perioperative management.

## 2. Results

### 2.1. Inflammation-Modulating Strategies in Abdominal and Liver Surgery

**Figure 2:**
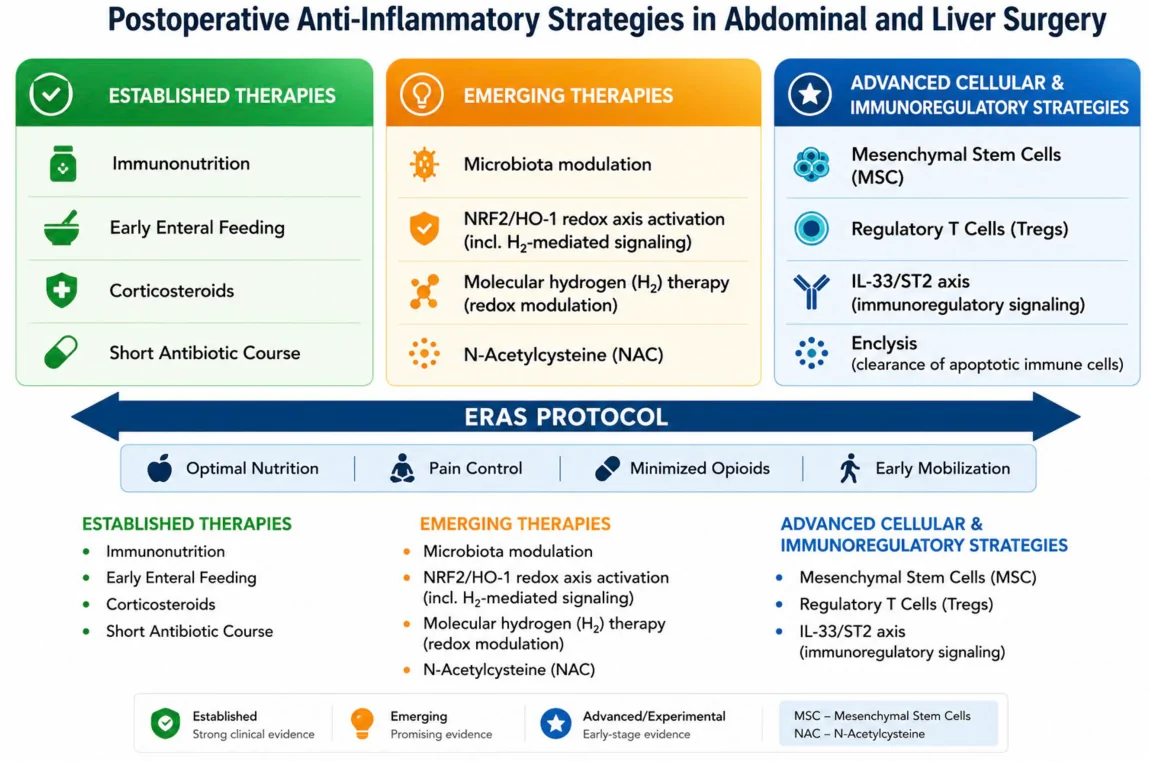
Spectrum of modern anti-inflammatory strategies after abdominal surgery: from immunonutrition to cellular therapies [1,2,3,6,10,14,16,21,22,23,24,34,35].

Over the past decade, the approach to the postoperative inflammatory response has undergone a substantial shift: rather than nonspecific immunosuppression, current strategies emphasize precise, targeted immunomodulation tailored to the patient’s surgical and metabolic profile. This contemporary paradigm integrates metabolic, oxidative, and cellular mechanisms within ERAS protocols, aiming to reduce inflammatory stress, maintain homeostasis, and accelerate postoperative recovery [2,5,25,26]. Core ERAS principles include early mobilization, optimal nutrition (with early enteral feeding), opioid minimization, effective pain control, and restricted fluid therapy [27,28,29]. Individualized perioperative care integrates nutritional, metabolic, and procedure-specific interventions to optimize postoperative recovery (Figure 2) [1,2,30,31,32,33].

Figure 2 presents a structured overview of contemporary inflammation-modulating strategies following abdominal and liver surgery, organized into three conceptual groups according to the nature and maturity of the supporting evidence and their stage of clinical translation. The figure provides a conceptual framework encompassing both established interventions and selected emerging approaches of translational relevance, thereby illustrating the continuum from evidence-based perioperative strategies to experimental molecular and cell-based therapies. The first group comprises clinically established perioperative interventions supported by randomized trials, meta-analyses, and/or guideline recommendations, including immunonutrition, early enteral feeding, perioperative corticosteroid therapy, and short-course antibiotic therapy following effective source control.

The second group comprises emerging and translational therapies targeting intestinal barrier integrity and redox homeostasis. These include microbiota modulation (e.g., probiotics and fecal microbiota transplantation), activation of the NRF2/HO-1 redox axis, and N-acetylcysteine (NAC).

Together, these approaches address oxidative stress and immune–metabolic dysregulation, which are particularly relevant in high-risk settings such as extensive liver resections or transplantation.

The third group encompasses advanced cellular and immunoregulatory strategies, including MSCs, Tregs, modulation of the IL-33/ST2 axis, and enclysis, the hepatocyte-mediated engulfment of live CD4+ T cells [14]. These experimental approaches target immune regulation and regeneration; the therapeutic implications of modulating enclysis remain to be established.

**Figure 3:**
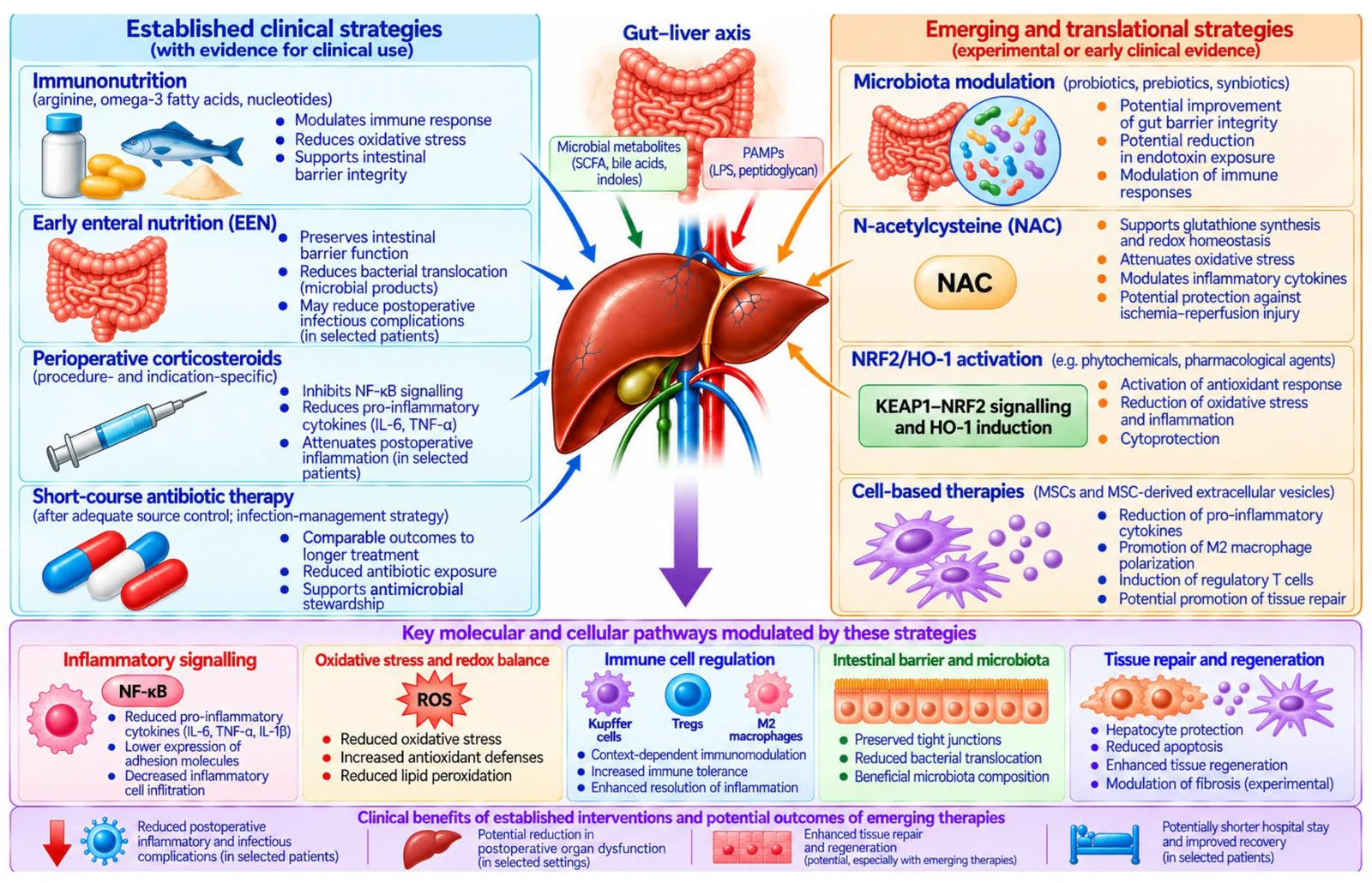
Molecular and cellular mechanisms of anti-inflammatory strategies after abdominal and liver surgery [1,3,6,10,14,16,17,18,21,22,23,24,36,37].

The molecular and cellular mechanisms underlying these strategies, including their effects on inflammatory signaling, oxidative stress, immune regulation, intestinal barrier integrity, and tissue repair, are summarized in Figure 3.

A meta-analysis of randomized controlled trials demonstrated that perioperative immunonutrition significantly reduced total postoperative complications (RR 0.78, 95% CI 0.66–0.93) and infectious complications (RR 0.71, 95% CI 0.61–0.82) compared with standard nutritional therapy [3].

A meta-analysis of 10 randomized controlled trials involving 930 patients demonstrated that perioperative corticosteroid administration significantly reduced overall postoperative complications (OR 0.61, 95% CI 0.43–0.87), postoperative serum bilirubin (MD −0.46, 95% CI −0.74 to −0.18), interleukin-6 concentrations (MD −48.99, 95% CI −60.72 to −37.27), and C-reactive protein levels (MD −5.20, 95% CI −7.62 to −2.77), without increasing infectious, thromboembolic, or bleeding complications [38]. A systematic review and meta-analysis of randomized controlled trials demonstrated that early oral feeding after upper gastrointestinal surgery significantly shortened the time to first flatus and length of hospital stay and reduced the risk of pneumonia, without increasing the risk of anastomotic leakage, reoperation, readmission, or mortality compared with delayed oral feeding [39]. Randomized trials and contemporary evidence-based guidelines demonstrate that, following adequate source control, short-course antibiotic therapy (≤4 days) is non-inferior to prolonged treatment with respect to recurrent infection and mortality, while significantly reducing antibiotic exposure, antimicrobial selection pressure, and disruption of the gut microbiota [22,23]. A systematic review and meta-analysis of randomized controlled trials demonstrated that perioperative probiotics and synbiotics significantly reduced postoperative infectious complications following colorectal surgery while improving recovery of intestinal barrier function; however, marked heterogeneity in probiotic strains, dosing regimens, and treatment protocols currently limits their routine implementation in all surgical patients [24].

In conditions characterized by high oxidative stress, such as liver resection or transplantation, redox-targeted interventions include NAC [34,36] and NRF2 pathway activation [21]. Modern anti-inflammatory strategies after abdominal surgery—mechanisms, evidence, and implementation—are summarized in Table 1. The comparative overview integrates findings from systematic reviews, meta-analyses, randomized controlled trials, and translational investigations, highlighting the biological mechanisms, clinical relevance, and varying degrees of evidence supporting established and emerging perioperative anti-inflammatory strategies.

While Table 1 summarizes the biological rationale and mechanisms of action of perioperative inflammation-modulating interventions, Table 2 complements this overview by presenting quantitative effect estimates reported in selected clinical evidence syntheses.

EEN initiated within 24 h after surgery is recommended by the European Society for Clinical Nutrition and Metabolism (ESPEN) as the standard of care in surgical practice, emphasizing early restoration of oral intake and avoidance of prolonged fasting. In the most recent ESPEN updates, nutritional management is highlighted as an integral component of ERAS and should be implemented systematically as part of a comprehensive perioperative strategy incorporating early nutrition, control of metabolic stress, and optimization of energy–protein balance [1,42,43,44]. When enteral nutrition alone does not meet energy and protein requirements, supplemental parenteral nutrition may be considered to prevent inadequate energy delivery, with the timing and extent of supplementation tailored to the patient’s nutritional risk and tolerance of enteral feeding [45,46]. The 2025 ESPEN guidelines emphasize early initiation of nutritional therapy when nutritional risk becomes apparent and recommend integrating oral, enteral, and parenteral nutrition according to individual nutritional requirements and the feasibility of achieving adequate intake [1]. Table 1 summarizes the principal perioperative anti-inflammatory interventions identified in the literature, including established nutritional strategies, microbiota-targeted approaches, redox-modulating therapies, and emerging cell-based immunomodulatory interventions. The included strategies are presented according to their principal biological targets, reported postoperative effects, characteristics and maturity of the supporting evidence, and potential relevance within ERAS-based perioperative care.

Immunonutrition and early enteral nutrition were the most consistently investigated perioperative interventions across the included randomized controlled trials and meta-analyses. Reported benefits included reductions in postoperative infectious complications, shorter hospital stays, and improved recovery parameters, although the effects on inflammatory biomarkers were less consistent across studies. Early enteral nutrition was consistently associated with lower rates of infectious complications compared with total parenteral nutrition and remains a key component of ERAS protocols.

Studies evaluating microbiota-targeted interventions, including probiotics, synbiotics, and fecal microbiota transplantation, reported potential benefits related to intestinal barrier function, immune regulation, and postoperative infectious complications [47]. However, considerable heterogeneity in probiotic strains, treatment protocols, patient populations, and clinical endpoints was consistently identified across studies, limiting direct comparison and routine clinical implementation. Studies investigating redox-targeted interventions, including N-acetylcysteine and NRF2/HO-1 pathway activation, primarily consisted of preclinical, translational, and early clinical studies. These interventions were associated with attenuation of oxidative stress, modulation of inflammatory signaling pathways, and potential organ-protective effects; however, randomized clinical trials evaluating postoperative clinical outcomes remain limited.

Evidence on MSC-based therapies, MSC-derived extracellular vesicles, and regulatory T cell-based approaches was derived predominantly from preclinical or translational studies, with limited clinical evidence available in abdominal surgery. Reported findings indicate anti-inflammatory and immunomodulatory effects mediated through macrophage polarization, increased IL-10 signaling, and regulation of the T helper 17 (Th17)/Treg balance. Clinical evidence remains largely confined to transplantation studies, and no randomized controlled trials have evaluated these approaches in the immediate postoperative setting following abdominal surgery. Overall, the reviewed studies encompass interventions ranging from established ERAS-based perioperative strategies to emerging molecular and cell-based approaches that are currently undergoing translational evaluation.

### 2.2. ERAS-Based Anti-Inflammatory Strategies in Abdominal and Liver Surgery

Current perioperative interventions increasingly focus on limiting excessive inflammatory responses and reducing the risk of complications following surgical procedures, including those within the abdominal cavity. Many of these strategies, supported by robust data from randomized trials, have been incorporated into contemporary ERAS pathways as components that demonstrably improve clinical outcomes. The principal benefits include a reduction in infectious complications, preservation of immunometabolic homeostasis, and a shorter recovery period. The reviewed evidence identified several perioperative strategies supported by comparatively robust clinical data, which are summarized in Table 3.

Immunonutrition containing arginine, ω-3 fatty acids, and nucleotides was associated with fewer infectious complications and shorter hospital stays, although effect sizes varied across studies [3,4,5]. Perioperative corticosteroid therapy in liver resections was associated with a marked decrease in IL-6 and CRP levels without an increased risk of infection. EEN may reduce mortality after emergency abdominal surgery, although the certainty of evidence remains low [6]. Short-course antibiotic therapy (≤4 days) following effective source control proved optimal, as it limited both antibiotic exposure and the risk of adverse effects (Table 3). Table 3 provides a structured comparison of perioperative anti-inflammatory strategies according to biological targets, clinical effects, and feasibility of integration within ERAS pathways. Several of the clinically established interventions summarized in Table 3 are implemented early in the perioperative course, underscoring the importance of timely management of nutritional, inflammatory, and infection-related risk. Immunonutrition and early enteral nutrition represent important components of multimodal perioperative care, although the magnitude and clinical relevance of their benefits may vary according to nutritional status, surgical complexity, patient risk profile, and the specific nutritional intervention applied. Their favorable safety profile and ease of implementation explain their consistent inclusion in ERAS and ESPEN recommendations across abdominal and liver surgery. Perioperative corticosteroids form a distinct category characterized by high biological potency but requiring strict control of timing and dosage, particularly in the context of hepatic resection. In elective liver resection, available randomized evidence indicates that short-course perioperative corticosteroid administration can attenuate the postoperative inflammatory response without a demonstrated increase in infectious complications, although optimal dosing, timing, and patient selection remain to be established. Following adequate source control of complicated intra-abdominal infection, evidence supports short-course antibiotic therapy as an effective alternative to prolonged treatment, reducing antimicrobial exposure without compromising relevant clinical outcomes. This finding highlights a shift toward inflammation-aware antimicrobial stewardship as an integral component of ERAS-based care. Redox-targeted interventions such as N-acetylcysteine have demonstrated effects on selected surrogate markers of oxidative and metabolic stress; however, these effects have not translated consistently into improvements in clinically meaningful postoperative outcomes. The currently limited and heterogeneous clinical evidence for redox-targeted interventions supports their consideration as investigational or adjunctive approaches rather than routine components of perioperative management. Microbiota-directed approaches occupy an intermediate position, supported by a strong mechanistic rationale but limited by heterogeneity in clinical protocols and patient selection. Their effects on intestinal barrier integrity and immune regulation provide a biological rationale for further investigation, but clinically meaningful patient-selection criteria and standardized therapeutic protocols remain to be established. Source control and structured sepsis management remain fundamental components of perioperative care and essential prerequisites for the appropriate application of adjunctive inflammation-modulating strategies. Importantly, Table 3 illustrates that the clinical relevance of individual interventions depends not only on the nature and consistency of the available evidence but also on the surgical context and timing of intervention.

The comparative structure of Table 3 therefore provides a framework for interpreting the potential clinical applicability of individual strategies in relation to the strength and consistency of the available evidence, surgical complexity, and perioperative timing.

## 3. Discussion

The evidence synthesized in this review illustrates the ongoing transition in perioperative management from generalized suppression of postoperative inflammation toward precision immunomodulation that integrates nutritional, pharmacological, metabolic, microbiota-targeted, and cell-based strategies within ERAS pathways. The integration of nutritional, pharmacological, and microbiota-targeted interventions within ERAS reflects a broader movement toward personalized medicine in surgery. Among clinically established perioperative strategies, immunonutrition and early enteral nutrition have been associated with reductions in postoperative infectious complications and improvements in recovery-related outcomes, whereas appropriately selected corticosteroid regimens and short-course antibiotic therapy may provide procedure- and indication-specific benefits within defined clinical settings. Conversely, therapies targeting redox balance and the microbiota (e.g., NRF2 activators and fecal microbiota transplantation) define an emerging domain within surgical immunology. Their full implementation will require further phase II/III studies assessing inflammatory biomarkers (IL-6, CRP, HO-1) and standardization of translational protocols [15,20,21,49,52].

EEN is among the most extensively investigated perioperative nutritional strategies and has been associated with reduced bloodstream infections, shorter intensive care unit and hospital stay, and improved recovery compared with early parenteral nutrition in critically ill adults [9].

Evidence indicates that early oral feeding after gastrointestinal surgery accelerates the return of bowel function and shortens hospital stay [41]. These findings are consistent with those reported by Deng et al. for upper gastrointestinal surgery [39]. Furthermore, the updated 2025 ERAS recommendations by Gustafsson et al. emphasize early oral feeding, minimization of decompression tubes, and implementation of the nudging protocol, highlighting the central role of nutritional strategies in modulating the postoperative response [2]. High compliance with ERAS recommendations has consistently been associated with shorter hospital stay, fewer postoperative complications, and improved metabolic recovery following abdominal surgery [53,54,55].

Although the available evidence is not entirely uniform, EEN appears preferable as the primary nutritional route because of its association with a lower risk of postoperative infection [6,9]. Importantly, Gao et al. demonstrated that early supplemental parenteral nutrition, administered when enteral nutrition alone was insufficient, significantly reduced hospital-acquired infections compared with late supplemental parenteral nutrition in adults undergoing major abdominal surgery [8]. These findings support a stepwise ERAS-based strategy in which enteral nutrition is prioritized, and parenteral supplementation is introduced early when energy and protein requirements cannot be adequately achieved through the enteral route.

In pancreatic surgery, ERAS recommendations also emphasize the benefits of early nutritional interventions, multimodal analgesia, and early mobilization, which collectively reduce complications and shorten hospital stay [56,57]. Prospective validation of ERAS recommendations in liver surgery likewise supports their clinical value in perioperative care [54]. Growing understanding of peripheral immune tolerance, particularly the central role of Tregs and the transcription factor FOXP3, has renewed interest in therapeutic strategies aimed at enhancing Treg-mediated immune regulation in the perioperative setting following abdominal surgery. Such an approach aims to limit excessive inflammatory responses and restore balance within the Th17/Treg axis. At the molecular level, this balance reflects opposing immunoregulatory programs: FOXP3-expressing Tregs suppress excessive immune activation through mechanisms including IL-10 and transforming growth factor-β signaling, whereas Th17 cells, driven by the transcription factor retinoic acid receptor-related orphan receptor γt, promote pro-inflammatory responses predominantly through interleukin-17 production; consequently, disruption of the Treg/Th17 equilibrium may contribute to persistent inflammatory signaling and impaired immune homeostasis. Mechanisms involving modulation of FOXP3 expression and restoration of the Th17/Treg balance may, in the future, support the development of personalized immunomodulatory strategies in surgical practice, integrating translational immunology with perioperative care pathways [40]. Further implementation of ERAS protocols in abdominal and liver surgery is increasingly shifting the focus from a “one-size-fits-all” approach toward risk stratification and individualized intervention selection based on inflammatory phenotype, nutritional risk, and tolerance of enteral feeding [1,2,30,58,59]. The most recent ERAS Society recommendations for colorectal surgery (2025) emphasize early feeding, avoidance of routine nasogastric decompression, and behavioral strategies such as “nudging” to improve protocol adherence, with these measures collectively contributing to reduced postoperative morbidity and shorter hospital stay [2]. With regard to immunonutrition, contemporary umbrella reviews and meta-analyses demonstrate a significant reduction in postoperative complications, particularly infectious events, in gastrointestinal oncologic surgery, while simultaneously highlighting heterogeneity in formula composition, timing of administration, and patient populations as the primary sources of variability in clinical effect [4]. Additional sources of heterogeneity include the duration of preoperative immunonutrition, baseline nutritional and sarcopenia status, and differences in surgical case-mix, all of which may influence the magnitude and clinical relevance of the observed effects.

From a clinical perspective, the available evidence suggests that the greatest benefit of immunonutrition is achieved in high-risk patients, particularly those with malnutrition, sarcopenia, or undergoing major abdominal procedures, whereas in low-risk populations, the benefits appear to be limited primarily to reductions in infectious complications [1,3,4,60].

Early enteral nutrition is supported by a strong biological rationale, particularly through preservation of intestinal barrier integrity and modulation of the gut–immune axis; however, evidence from emergency abdominal surgery remains limited by methodological heterogeneity and variable certainty, underscoring the need for adequately powered trials with standardized outcome definitions and clinically relevant endpoints [6].

Concurrently, hybrid nutritional strategies that prioritize enteral nutrition while introducing early supplemental parenteral nutrition when nutritional targets cannot be achieved are gaining increasing clinical acceptance, particularly in high-risk surgical patients [8,45,46]. Regarding postoperative oral feeding after gastrointestinal surgery, recent systematic reviews confirm accelerated recovery of bowel function and reduced length of stay, while also emphasizing substantial heterogeneity in feeding protocols, including timing of initiation and diet composition, which necessitates cautious interpretation and further standardization [39,41]. In the domain of antimicrobial therapy following effective source control, current evidence-based guidelines support short-course regimens (≤4 days) as clinically non-inferior to prolonged treatment, while simultaneously reducing antibiotic exposure and selective pressure on the gut microbiota [22]. Complementary expert consensus documents on intra-abdominal infections further emphasize the need to integrate shortened antibiotic courses with close monitoring of inflammatory response and prioritization of timely and adequate source control [23]. With respect to gut microbiota modulation, meta-analyses of randomized controlled trials in colorectal surgery suggest a potential reduction in postoperative infectious complications with probiotics or synbiotics; however, the absence of consensus regarding optimal strains, dosing, and timing currently precludes their unequivocal incorporation into standard ERAS pathways [24].

In liver surgery, the close interplay between inflammation, oxidative stress, and ischemia–reperfusion injury renders redox-targeted interventions conceptually attractive; nevertheless, even the most recent meta-analyses evaluating N-acetylcysteine after hepatic resection fail to demonstrate consistent improvement in key clinical outcomes, providing no basis for routine use after liver resection [34]. In parallel, pharmacological activation of the NRF2/HO-1 axis is emerging as a mechanistically compelling cytoprotective strategy, with translational evidence supporting its potential to attenuate oxidative stress, inflammatory signaling, and cell injury and providing a rationale for dedicated perioperative studies in abdominal and liver surgery [21]. Additionally, mesenchymal stem cell-derived extracellular vesicles are being investigated as potential modulators of inflammatory responses, with preclinical and translational evidence supporting further evaluation of their therapeutic potential in inflammatory conditions [15].

Future research should prioritize adequately powered phase II/III randomized trials in major abdominal and liver surgery that integrate standardized longitudinal assessment of inflammatory and immunometabolic biomarkers with clinically meaningful, organ-specific endpoints, while prospectively evaluating the predictive and treatment-monitoring performance of candidate markers such as IL-6, CRP, and HO-1 [36,52]. In parallel, emerging MSC-based therapeutic strategies warrant further investigation in liver-related inflammatory and regenerative settings, with future studies needed to clarify their clinical efficacy, mechanisms of action, and safety [16].

Moreover, accumulating preclinical and translational evidence indicates that the immunomodulatory effects of MSCs are mediated, at least in part, through paracrine mechanisms rather than durable tissue engraftment, with extracellular vesicles increasingly recognized as important mediators of these effects [17]. MSC-derived extracellular vesicles have been shown to modulate innate and adaptive immune responses by promoting macrophage polarization toward an anti-inflammatory M2 phenotype, suppressing NF-κB signaling and NLR family pyrin domain containing 3 inflammasome activation, and facilitating the expansion of regulatory T cells. These immunomodulatory effects are mediated, at least in part, by the transfer of bioactive extracellular vesicle cargo—including microRNAs, proteins, and other regulatory molecules—to recipient immune and parenchymal cells, thereby modifying intracellular signaling pathways involved in inflammatory activation, cell survival, and tissue repair. Collectively, these effects contribute to attenuation of systemic postoperative inflammation in experimental models of surgery and ischemia–reperfusion injury [18]. Importantly, cell-free therapeutic strategies based on MSC-derived vesicles may offer potential safety advantages over live-cell therapies by avoiding risks associated with cellular engraftment and uncontrolled persistence, although their safety profile requires further validation in well-designed clinical studies [19]. Despite their promising immunomodulatory potential, experimental approaches such as fecal microbiota transplantation and MSC-derived exosome-based therapies are associated with important limitations and risks in the postoperative setting. These include safety concerns related to infection transmission, immunological unpredictability, lack of standardized preparation and dosing protocols, and limited clinical evidence supporting their routine use, which currently restricts their application to controlled research settings. Recent translational and clinical literature emphasizes the need for standardized extracellular vesicle isolation and characterization, harmonized dose metrics, and consistent outcome reporting to improve reproducibility and facilitate clinical translation of MSC-derived extracellular vesicle therapies [20].

From a health system perspective, established perioperative strategies such as immunonutrition, early enteral nutrition, and short-course antibiotic therapy are generally cost-effective, as they are associated with reduced complication rates, shorter hospital stays, and lower overall resource utilization [61]. In contrast, emerging interventions, including advanced microbiota-based or cell-derived therapies, may entail higher up-front costs and logistical complexity, underscoring the need for formal cost-effectiveness analyses before their routine implementation in clinical practice.

Despite the growing body of evidence supporting several perioperative immunomodulatory strategies, this review has several limitations that should be acknowledged. First, the heterogeneity of study designs, patient populations, and intervention protocols limits direct comparability of results and precludes quantitative synthesis [62]. Second, a number of included studies reported neutral or inconsistent effects on inflammatory biomarkers or clinical outcomes, particularly in low-risk patient populations, which underscores the need for cautious interpretation of overall conclusions. Finally, variability in outcome definitions and follow-up duration across studies may have contributed to differences in reported efficacy, highlighting the necessity for standardized endpoints in future trials [63,64]. Nevertheless, the available evidence supports a perioperative approach centered on controlled modulation, rather than indiscriminate suppression, of the inflammatory response, consistent with contemporary principles of enhanced recovery and organ protection in abdominal and liver surgery.

Furthermore, because this work was designed as a narrative review supported by a structured literature search rather than as a systematic review, no formal risk-of-bias assessment or certainty-of-evidence grading framework was applied; consequently, the evidence profiles presented herein should be interpreted as descriptive summaries of the type, consistency, and translational maturity of the available evidence rather than as formal evidence-certainty ratings.

**Figure 4:**
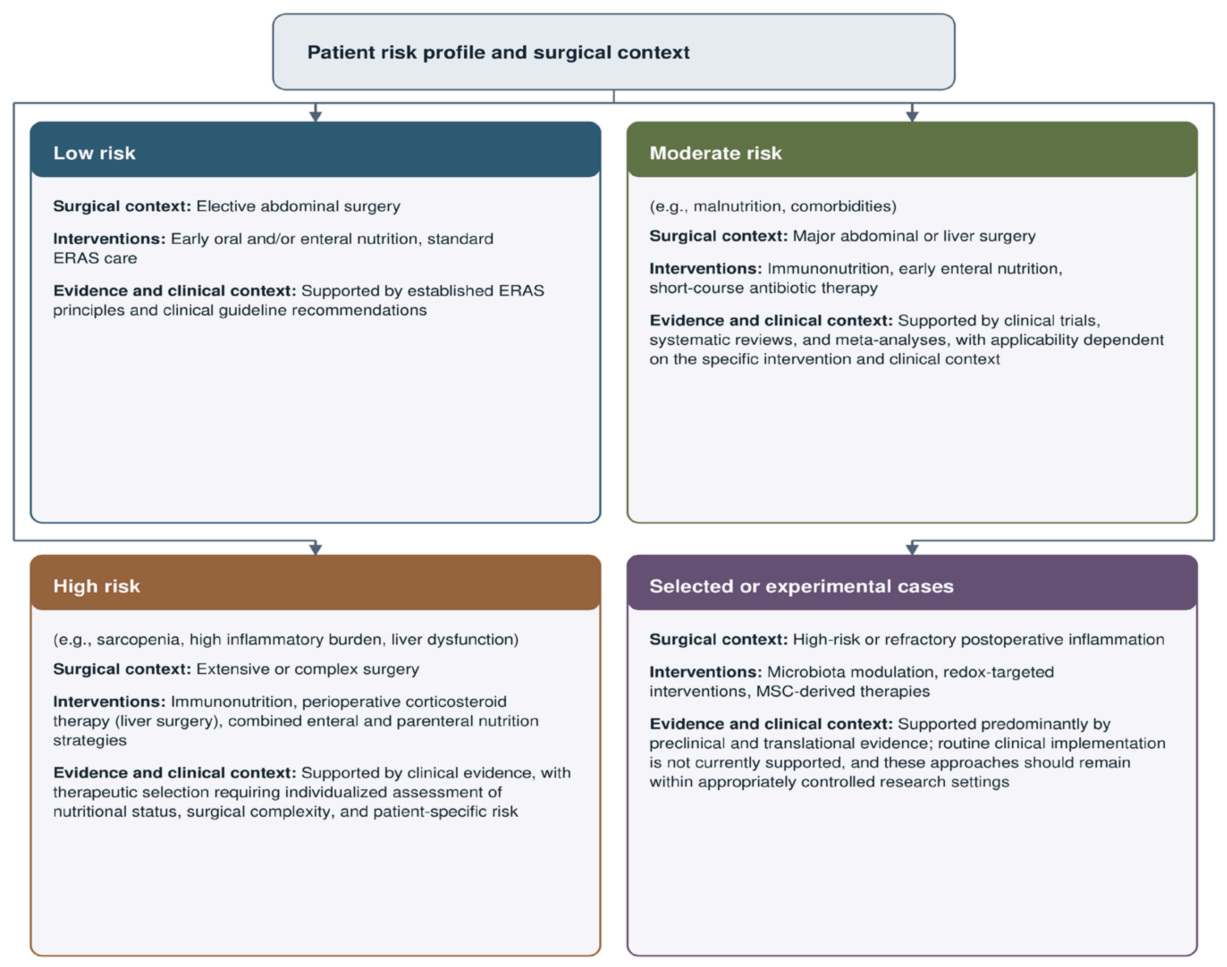
Practical, stepwise approach to perioperative anti-inflammatory interventions based on patient risk profile and surgical context [1,2,3,4,5,6,8,10,15,16,17,18,19,20,21,22,23,24,34,35,36,39,41,45,46,47,49].

To facilitate clinical interpretation and translational applicability of the discussed evidence, a schematic summary (Figure 4) outlining a stepwise approach to the selection of perioperative anti-inflammatory interventions based on patient risk profile and surgical context is provided below.

Future clinical trials should incorporate standardized biomarker panels and predefined longitudinal sampling protocols to determine whether dynamic inflammatory and immunometabolic profiles can reliably characterize treatment response and contribute to personalized perioperative management [65].

Serial assessment of inflammatory and immunometabolic markers, including IL-6, CRP, and HO-1, warrants further prospective evaluation to determine their potential utility for monitoring treatment response and perioperative risk stratification, particularly with respect to predictive performance, assay standardization, and optimal sampling time points. However, the current evidence does not establish sufficiently standardized sensitivity, specificity, predictive thresholds, or sampling schedules for these markers to support their routine use for perioperative risk stratification or treatment monitoring [36,52].

The close integration of hepatic immune regulation and nutrient metabolism is further illustrated by studies of physiological adaptations during pregnancy, which demonstrate the liver’s capacity to coordinate immunological responses and glucose, lipid, and protein metabolism under changing metabolic demands [66,67]. Although these findings arise from a distinct physiological context, they reinforce the broader concept of hepatic immunometabolic integration relevant to understanding systemic responses to physiological stress.

## 4. Materials and Methods

This narrative review was conducted using a structured literature search to support a critical and integrative synthesis of clinical and translational evidence on perioperative strategies modulating postoperative inflammation after abdominal and liver surgery.

The PubMed, Scopus, Web of Science, and Cochrane Library databases were searched using a structured approach for studies published between 2015 and 2025. The search strategy included combinations of keywords related to postoperative inflammation and perioperative care, including abdominal surgery, liver surgery, postoperative inflammation, immunonutrition, perioperative corticosteroids, early enteral nutrition, short-course antibiotics, microbiota modulation, NRF2, molecular hydrogen therapy, and MSC-derived exosomes. Search terms were combined using the Boolean operators AND/OR, with database-specific adaptations where required.

The literature considered for this review focused primarily on studies involving adult patients undergoing abdominal and/or liver surgery and reporting clinically or translationally relevant evidence on postoperative inflammation, perioperative interventions, inflammatory biomarkers, or related clinical outcomes. Pediatric studies, case reports, preclinical investigations without clear translational relevance, and publications that did not provide information pertinent to the scope of the review were not considered in the narrative synthesis.

The identified literature was critically and narratively synthesized with consideration of study design, methodological robustness, clinical relevance, consistency of reported findings, and the biological plausibility of the proposed mechanisms. Greater interpretative weight was given to current clinical guidelines, systematic reviews, meta-analyses, and randomized controlled trials, whereas observational, translational, and preclinical studies were primarily used to contextualize clinical findings, elucidate molecular mechanisms, and discuss emerging therapeutic approaches. Given the substantial heterogeneity in study populations, interventions, outcome measures, and study designs, a de novo quantitative synthesis was not undertaken. Accordingly, the evidence profiles presented in Table 1, Table 2 and Table 3 and Figure 4 were used descriptively to characterize the design, consistency, clinical directness, and translational maturity of the supporting literature and should not be interpreted as formal certainty-of-evidence ratings.

## 5. Conclusions

Current clinical evidence supports a multimodal approach to perioperative inflammation management, integrating established nutritional strategies such as immunonutrition and early enteral nutrition with procedure- and indication-specific interventions, including perioperative corticosteroid therapy in liver resection and short-course antibiotic therapy following adequate source control.

Emerging approaches targeting the gut microbiota and redox homeostasis warrant further investigation in well-designed clinical trials incorporating standardized interventions, clinically meaningful endpoints, and prospective validation of candidate biomarkers to determine their potential role in patient stratification and individualized perioperative management.

## Figures and Tables

**Table 1:** Anti-inflammatory strategies in abdominal and liver surgery.

Module/Intervention	Molecular Target/System	Main Clinical Effects	Evidence Profile	ERAS Recommendation	Ref.
Immunonutrition (arginine, ω-3 fatty acids, nucleotides)	↓ IL-6/CRP, PGE2 modulation, T-cell activation	↓ infectious complications; ↓ hospital stay; inflammatory biomarkers—mixed results	Supported by systematic reviews, meta-analyses, and randomized controlled trials	Preferred in high-risk patients; start 5–7 days preoperatively and continue postoperatively	[1,2,3,4,5,6]
Early enteral nutrition (EEN)	Gut barrier preservation; gut–immune axis	↓ infections vs. TPN; similar non-infectious complications	Supported by systematic reviews, meta-analyses, and randomized controlled trials	Start ≤ 24 h postoperatively, if feasible	[1,6,9]
Microbiota modulation (probiotics/synbiotics)	Treg/FOXP3 pathway; gut integrity	Potential ↓ mucosal inflammation; immune regulation	Supported by translational studies and emerging clinical evidence	Consider in selected dysbiosis; standardized protocols required	[24]
NRF2 activation	NRF2 pathway	↓ oxidative stress; potential cytoprotective effects	Supported primarily by translational and ex vivo evidence	Candidate for future clinical translation	[21]
Mesenchymal stem cells (MSCs)/MSC-derived exosomes	Macrophage polarization (M2); ↑ IL-10	Anti-inflammatory and regenerative effects	Supported primarily by preclinical and translational evidence	Experimental therapy only	[15,16,17]
Treg-based therapy/Th17–Treg modulation	Immunotolerance; Th17/Treg balance	Potential improvement of graft immune tolerance	Supported primarily by preclinical, translational, and transplantation evidence	Potential future application in transplantation	[35,40]

Abbreviations: IL-6, interleukin-6; CRP, C-reactive protein; PGE2, prostaglandin E2; EEN, early enteral nutrition; TPN, total parenteral nutrition; Treg, regulatory T cell; FOXP3, forkhead box P3; NRF2, nuclear factor erythroid 2-related factor 2; MSC, mesenchymal stem cell; Th17, T helper 17 cell; IL-10, interleukin-10.

**Table 2:** Quantitative effect estimates reported in selected clinical evidence syntheses.

**Intervention**	**Study Design**	**Main Quantitative Findings**	**Clinical Interpretation**	**Ref.**
Perioperative immunonutrition (arginine, ω-3 fatty acids, nucleotides)	Systematic review and meta-analysis of randomized controlled trials	Overall postoperative complications: RR 0.78 (95% CI 0.66–0.93); infectious complications: RR 0.71 (95% CI 0.61–0.82)	Significantly reduces overall and infectious postoperative complications.	[3]
Perioperative corticosteroids (elective liver resection)	Meta-analysis of 10 randomized controlled trials (930 patients)	Overall complications: OR 0.61 (95% CI 0.43–0.87); IL-6: MD −48.99 (95% CI −60.72 to −37.27); CRP: MD −5.20 (95% CI −7.62 to −2.77); bilirubin: MD −0.46 (95% CI −0.74 to −0.18)	Reduces postoperative inflammatory response without increasing infectious or bleeding complications.	[38]
Early enteral nutrition after emergency abdominal surgery	Systematic review and meta-analysis with trial sequential analysis	Mortality: OR 0.59 (95% CI 0.34–1.00)	Associated with lower postoperative mortality, although certainty of evidence remains low.	[6]
Early oral feeding after gastrointestinal surgery	Systematic review and meta-analysis of 34 randomized controlled trials	First bowel movement: MD −0.99 days (95% CI −1.25 to −0.72); first flatus: MD −0.70 days (95% CI −0.87 to −0.53); length of hospital stay: MD −1.31 days (95% CI −1.59 to −1.03); postoperative complications: RR 0.69 (95% CI 0.59–0.80)	Accelerates gastrointestinal recovery, shortens hospitalization and reduces postoperative complications without increasing vomiting.	[41]
Short-course antibiotic therapy after adequate source control	Systematic review and international guideline	Recommended duration: ≤4 days after definitive source control; prolonged therapy provides no additional clinical benefit.	Supports antimicrobial stewardship while maintaining clinical effectiveness.	[22,23]
Perioperative probiotics/synbiotics in colorectal surgery	Systematic review and meta-analysis of 21 randomized controlled trials	Overall infectious complications: RR 0.59 (95% CI 0.47–0.75); surgical-site infection: RR 0.70 (95% CI 0.52–0.95); respiratory infections: RR 0.35 (95% CI 0.20–0.63); urinary tract infections: RR 0.41 (95% CI 0.19–0.87)	Significantly reduces postoperative infectious complications; routine implementation remains limited by heterogeneity of probiotic preparations.	[24]
N-acetylcysteine after liver resection	Systematic review and meta-analysis	Postoperative lactate: weighted mean difference −0.61 (95% CI −1.19 to −0.04), indicating a statistically significant reduction; however, no significant improvement was observed in postoperative liver function, morbidity, or length of hospital stay.	Current evidence does not support routine use after liver resection.	[34]

Abbreviations: CI, confidence interval; CRP, C-reactive protein; IL-6, interleukin-6; MD, mean difference; OR, odds ratio; RR, risk ratio.

**Table 3:** Anti-inflammatory strategies after liver resection and abdominal surgery: clinical efficacy, modulatory targets, and integration with ERAS.

Intervention/Strategy	Biological Targets	Clinical Signals	Evidence Profile	Practical Conclusions	Ref.
Immunonutrition (arginine, omega-3, nucleotides)	Immunomodulatory effects, including changes in immune-cell function and inflammatory signaling	Reduced infectious complications in many studies; effects on inflammatory biomarkers are less consistent	Supported by multiple randomized controlled trials and meta-analyses	ERAS/ESPEN standard; pre- and postoperative use; best in malnourished/high-risk patients	[3,4,5,48]
Perioperative steroids (hydrocortisone, dexamethasone)	↓ IL-6/CRP; ↓ oxidative stress	↓ IL-6, CRP; better liver function; no ↑ infections	Supported by randomized controlled trials and meta-analyses	Short-term use after major hepatectomy; dose/timing to standardize	[10,38]
N-acetylcysteine (NAC)	↓ oxidative & reperfusion injury	↓ lactate; no clear effect on ALT/INR	Supported by systematic review and meta-analysis, with limited evidence of clinical benefit	Possible adjunct in large hepatectomies; not for routine use	[34]
Gut microbiota therapy (FMT, pre-/probiotics)	↑ barrier integrity; Treg/Th17 modulation; ↓ endotoxemia	↑ mucosal integrity; ↑ Treg/Th17; ↓ endotoxins	Supported by translational studies and emerging clinical evidence, with substantial heterogeneity across interventions and protocols	Promising in dysbiosis; needs standardized protocols	[24,49]
Short-course antibiotics	Limit sepsis/SIRS with minimal exposure	≤4 days as effective as longer courses following adequate source control	Supported by randomized clinical evidence and evidence-based international guidelines	Standard after adequate source control; avoid prolonged antibiotic therapy	[22,23]
Source control + sepsis management	↓ IL-6, TNF-α; metabolic stabilization	Improved parameters	Supported by clinical guidelines and observational evidence	Timely source control in abdominal sepsis	[23,50]
Early enteral nutrition (EEN)	Preserve gut integrity; ↓ IL-6	Possible ↓ mortality; low-certainty evidence	Supported by randomized controlled trials and meta-analyses, including trial sequential analysis	ERAS cornerstone; start ≤ 24 h	[1,6,9]
Multimodal opioid-sparing analgesia	Pain and surgical stress; ↓ opioid exposure	Improved analgesia; procedure-specific recovery benefits	Procedure-specific clinical evidence and recommendations	Select according to procedure and patient risk	[30,51]

Abbreviations: ERAS, Enhanced Recovery After Surgery; ESPEN, European Society for Clinical Nutrition and Metabolism; IL-6, interleukin-6; CRP, C-reactive protein; NAC, N-acetylcysteine; ALT, alanine aminotransferase; INR, international normalized ratio; FMT, fecal microbiota transplantation; Treg, regulatory T cell; Th17, T helper 17 cell; SIRS, systemic inflammatory response syndrome; EEN, early enteral nutrition.

## Data Availability

The original contributions presented in this study are included in the article. Further inquiries can be directed to the corresponding author.

## Abbreviations

The following abbreviations are used in this manuscript:

IL-6	Interleukin-6
IL-1β	Interleukin-1 beta
IL-10	Interleukin-10
TNF-α	Tumor necrosis factor-alpha
PGE2	Prostaglandin E2
NF-κB	Nuclear factor kappa-B
HMGB1	High-mobility group box 1
DAMP	Damage-associated molecular pattern
Treg	Regulatory T cell
Th17	T helper 17 cell
FOXP3	Forkhead box P3
NRF2	Nuclear factor erythroid 2-related factor 2
HO-1	Heme oxygenase-1
EEN	Early enteral nutrition
TPN	Total parenteral nutrition
FMT	Fecal microbiota transplantation
MSC	Mesenchymal stem cell
CRP	C-reactive protein
ERAS	Enhanced Recovery After Surgery
